# Validation of a pregnancy planning measure for Arabic-speaking women

**DOI:** 10.1371/journal.pone.0185433

**Published:** 2017-10-23

**Authors:** Eman Almaghaslah, Roger Rochat, Ghada Farhat

**Affiliations:** 1 Primary Health Care Administration and Preventive Health Department in Qatif, Saudi Arabian Ministry of Health, Qatif, Eastern province, Saudi Arabia; 2 Hubert Department of Global Health, Emory University, Atlanta, Georgia, United States of America; Weill Cornell Medical College, QATAR

## Abstract

**Background:**

The prevalence of unplanned pregnancy in Saudi Arabia has not been thoroughly investigated.

**Objective:**

To conduct a psychometric evaluation study of the Arabic version of the London Measure of Unplanned Pregnancy (LMUP).

**Methods:**

To evaluate the psychometric properties of the LMUP, we conducted a self-administered online survey among 796 ever-married Saudi women aged 20–49 years, and a re-test survey among 24 women. The psychometric properties evaluated included content validity measured by content validity index (CVI), structural validity assessed by exploratory factor analysis (EFA), substantive validity assessed by hypothesis testing, contextual stability for the test-retest assessed by weighted Kappa, and internal consistency assessed by Cronbach’s alpha.

**Results:**

The psychometric analysis of the Arabic version of LMUP exhibited valid and reliable properties. The CVIs for individual items and at the scale level were >0.7. EFA confirmed a unidimensional extraction of the scale item. Hypothesis testing confirmed expected associations. The tool was stable with weighted kappa = 0.78 and Cronbach’s alpha = 0.88.

**Conclusion and recommendations:**

In this study, the validity and reliability of the Arabic version of the LMUP were confirmed according to well-known psychometric criteria. This LMUP version can be used in research studies among Arabic-speaking women to measure unplanned pregnancy and investigate correlates and outcomes related to unplanned pregnancy.

## Introduction

Unplanned pregnancy, also referred to as mistimed, unintended, or unwanted pregnancy, has received a remarkable amount of attention from public health professionals and social scientists. Public health professionals use unintended pregnancy as a proxy for understanding population fertility and family planning (FP), and to measure the unmet need for contraception [1, 2]. Also, the reduction of unplanned pregnancy has become a key objective for many developed countries, including the United States (US) [2, 3] and the United Kingdom (UK) [4], as well as for global health organizations such as the United Nations Population Fund (UNFPA), which supports family planning interventions that aim to ensure that every pregnancy is wanted [5]. Moreover, social scientists have become more interested in studying unintended pregnancy as it has been linked to women’s autonomy–their ability to decide to have a baby or not and to choose the appropriate time for them–and to couples’ failure regarding contraceptive use, which might be because of unmet need for family planning, incorrect use of contraception, or absence of women’s agency in practicing reproductive rights [1].

**Fig 1:**
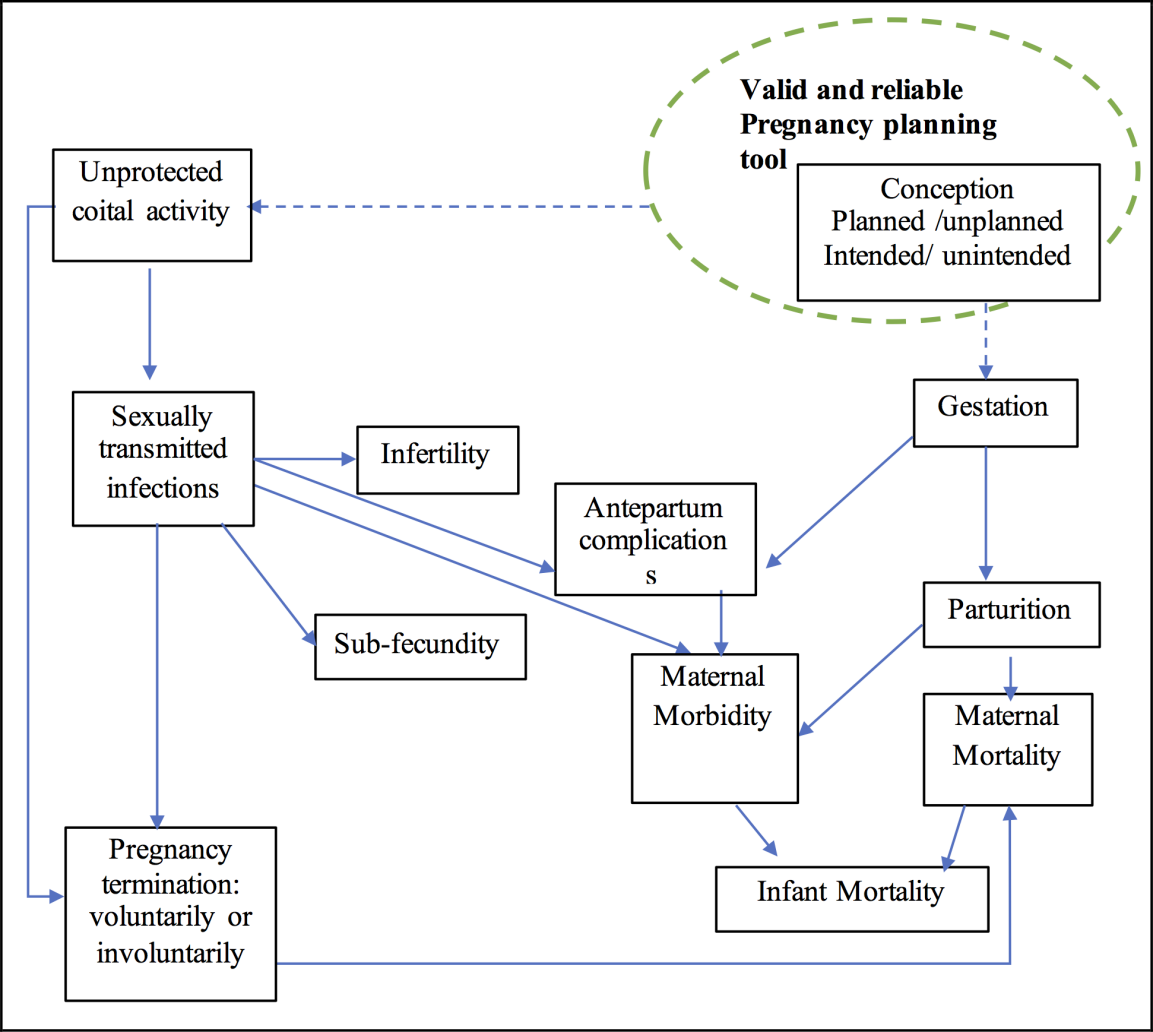
Pathways* for the impact of unplanned pregnancy on maternal and newborn outcomes *[9] → established correlations from previous literature by previous survey tools. ---The need for validation of the pregnancy planning measure to establish the relationships.

Unintended pregnancies may occur for several reasons which include: the incorrect use of contraception; the use of unreliable contraception method; personal factors, such as ambivalence in deciding whether to have a new child or not; health-related reasons; contraceptive side effects; lack of awareness and knowledge; cultural factors; or partner rejection [6, 7]. Unwanted pregnancies may adversely affect maternal and fetal health due to unsafe abortion[8] delayed antenatal care [9], adverse life outcomes for offspring [10], or reduced educational opportunities and financial situation for the woman as shown in Fig 1 [11–13].

Unintended pregnancy is difficult to measure[11] and different tools have been used to measure it. Most tools were not validated and assumed that pregnancy planning is a self-evident event [12]. Some used a single question format, which is not reliable to measure unplanned/unintended pregnancy [9, 12] [S1 Table]. Unplanned pregnancy should be measured through a valid tool to have scientific and clinical relevance. Barrett et al. investigated this in 2004, and developed a validated tool that addresses the complexity of pregnancy planning on a 6-item scale [12]. This measure, known as the London Measure of Unplanned Pregnancy (LMUP) [13]. The scale’s simplicity led to its application in multiple countries, including the United States, Malawi, Iran, India, and Brazil, where it was translated and validated using the native languages of those countries [7–11]. The scale’s world-wide usage confirmed that it is a simple and reliable method for assessing unplanned pregnancies. The LMUP scale measures the circumstances around a woman’s most recent pregnancy in regards to contraceptive use, timing of pregnancy, intention, desire to have a baby, discussion with the partner, and preconception preparation.

The issue of unplanned pregnancy in Arabic-speaking countries within the Middle East and North Africa (MENA) has rarely been investigated, partly due to the challenges in defining and measuring unplanned pregnancy [14]. Results from household surveys including the Pan Arab Project for Family Health (PAPFAM) and the Demographic and Health Survey (DHS) in six countries (Algeria, Lebanon, Morocco, Palestine, Syria, and Yemen) indicated that these countries have 1.2 million unintended births [1]. In Saudi Arabia, a study among postpartum women identified via convenience sampling estimated that 53% of women had unintended pregnancies. This estimate defined unintended pregnancy as a mistimed or unplanned pregnancy [15], but no clarification was provided about the tool that used to assess unintended pregnancy.

The scant data on unplanned pregnancy and the lack of a standard definition and method of assessment in Arabic-speaking countries constitute a major gap in our understanding of this important public health issue. Developing valid data to guide policy would improve maternal and child policies and health outcomes in these countries.

Therefore, this study aimed to develop an Arabic version of the LMUP and test its psychometric properties in Arabic-speaking women. The implications of this study are to facilitate future research on pregnancy planning and intention in Arabic-speaking nations.

## Methodology

### Population and sample

The study was approved by Emory University’s Institutional Review Board (IRB) following an expedited review (IRB approval number IRB00093915). All participants checked “agree” on a written consent to participate in the study. On a self-administered online survey of the Arabic piloted version of LMUP which conducted in April 2017. Among Arabic-speaking Saudi women who were ever-pregnant and of childbearing age (15–49 years). Social media platforms (e.g. WhatsApp) were used to recruit study participants using a non-probability sampling design.

Sample size calculation estimated that 383 responses are required for this study. The assumptions for sample size calculation included a 53.5% prevalence of unplanned pregnancy in Saudi Arabia [15], a confidence level of 95%, a design effect of 1, and a 2016 population estimate of 5,950,182 for ever-married Saudi women of childbearing age (15–49 years old) [16].

### Instrument

The six items of LMUP were tailored to Arabic-speaking Saudi women. The questionnaire included sociodemographic questions intended for hypothesis testing and to assess the respondents’ diversity and representation of the desired population. These questions included age, level of education (primary school, intermediate school, secondary school, diploma, bachelor, and post-graduate), area of residence (urban or rural), region in Saudi Arabia, number of pregnancies, number of children, whether the respondent is currently pregnant or not (gestational age if pregnant), whether currently lactating (postpartum) or not and age of the last child.

The LMUP 6 items (questions), each question scored as 0,1, 2, and the total LMUP score ranged from 0–12. The higher the score, the higher the pregnancy planning and intention. By avoiding dichotomous answers in the scoring of this measure, such as “planned” and “unplanned”, the LMUP makes it possible to assess ambivalence in pregnancy intention and understand its associated sociodemographic correlates [17, 18]. The LMUP scoring allows for an individual characterization of the full range of the scores. According to Barrett [19], a score cut-point of less than 3 indicates unplanned pregnancy, more than 10 indicates planned pregnancy, and scores of 4–9 indicate ambivalence of planning pregnancy [12].

### Procedures

The principal investigator (PI) who is native Arabic speaker and fluent in English, translated the LMUP into Arabic, prepared and conducted the phone interviews for piloting, then prepared the online questionnaire for dissemination (S1 File) and (S2 File). The PI preceeded the questionnaire by a consent form that describes the study purpose, target population, and participants’ rights.

The Arabic version of the LMUP was then piloted among five Saudi women who were eligible for study participation; they were contacted by phone and consented verbally to evaluate wording of the questionnaire and its acceptance (for the result and wording modification attached (S3 File)). Following the pilot, to further review the survey for content, a field epidemiologist from the Hubert Department of Global Health at Emory provided additional feedback. This epidemiologist had worked extensively in Arab settings and has familiarity with Arab culture.

The tool was modified based on recommended changes and was back-translated to English by an official translation services office in Saudi Arabia. The principal investigator ensured that the back translation was consistent in structure and content with the original version of LMUP.

The Arabic survey was disseminated electronically via social media platforms (Facebook, Twitter, and WhatsApp) to obtain a psychometric evaluation of the LMUP for Arabic speaking women. Electronic surveys were chosen for faster, long-distance, and anonymous data collection. Data was collected between February 11–22, 2017.

A total of 962 women responded to the survey within a period of 11 days. The responses of twelve women were excluded as they never had a pregnancy and who were not currently pregnant, because the LMUP is designed for retrospective measurement of pregnancy planning status [20].

**Fig 2:**
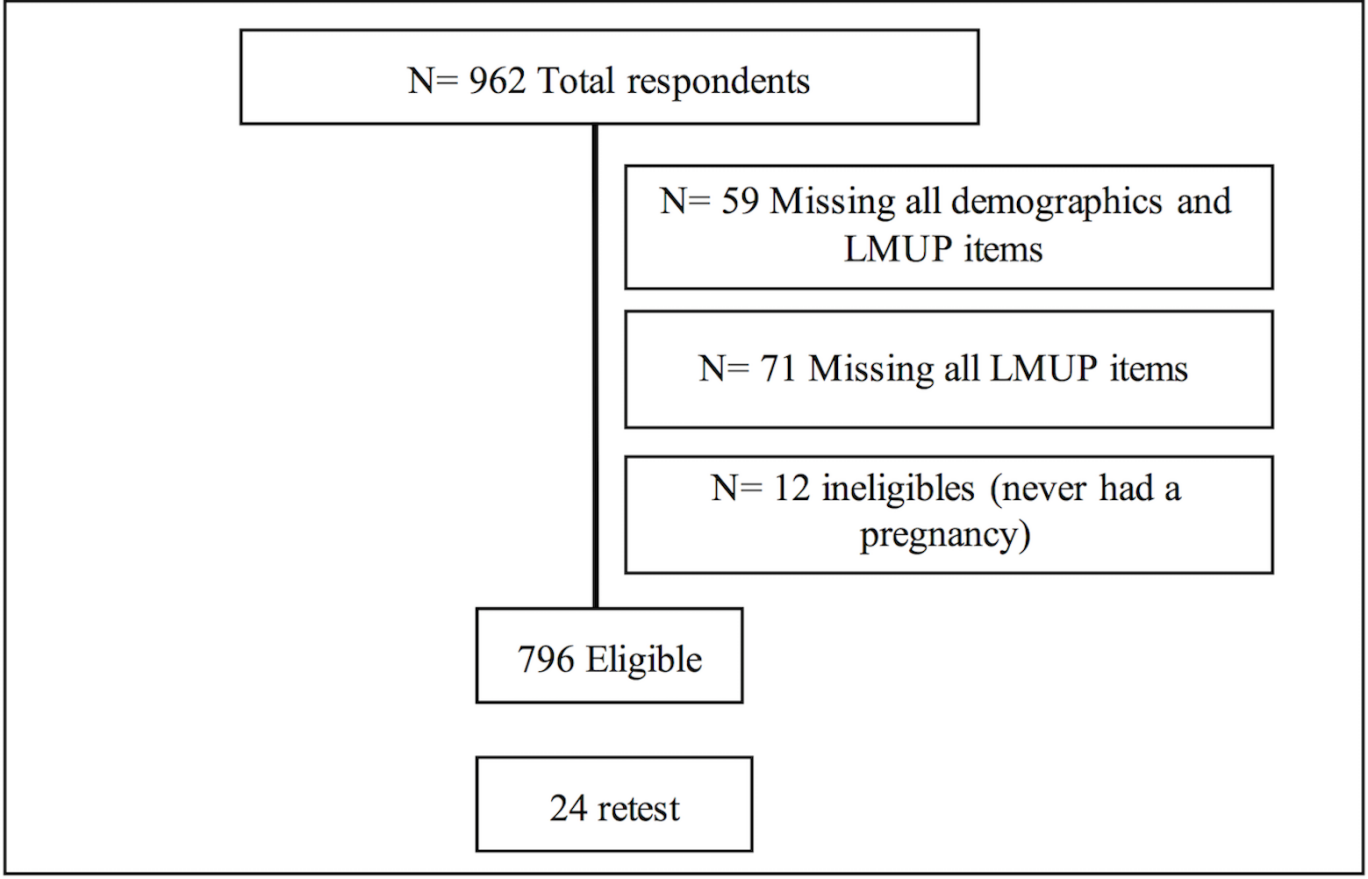
Participant flow chart.

Women were re-contacted after a period of 7 days and asked to retake the survey for test-retest assessment. A total of 24 women did the retest. A participants’ flowchart is presented in Fig 2.

To assess content validity, another online questionnaire was developed for specialists in family planning (FP) to rate the LMUP questions according to relevance in the Arabic context. The rating was structured on a four-point Likert scale (totally relevant, relevant, slightly relevant, not relevant).

### Psychometric assessment and statistical analysis

The analysis of the psychometric properties of the Arabic LMUP was done through the content, structural, substantive, as well as the generalizability aspects of validity [21].

*A. Content validity:* is “the ability of an instrument to reflect the domain of interest and the conceptual definition of the construct” [22]. The scale content relevance and representation to the pregnancy planning is determined by face validity, and technical quality of the items and their relationship to the content validity. Evidence of face validity and technical quality of the items determine content validity. Face validity achieves the acceptability of the tool in the context of a woman’s country [22]. We established face validity by pilot-testing the Arabic version of the LMUP in a group of five Saudi women and modifying the wording of the instrument according to the results of the pilot. The technical quality of the tool was established by using experts’ judgment i.e. FP experts. Also, social media platforms were used to identify and reach out to specialists. Twelve specialists rated the items, two of which were excluded (one was a general surgeon and not a FP expert, and the other provided ratings but commented that he/she did not understand the required task). Expert ratings provided feedback regarding suitability and the compatibility of the items in the validated measure [23].

We measured the content validity index for individual items (I-CVI) and for the whole scale (S-CVI). For I-CVI, the Likert scale (scored as 4 = totally relevant, 3 = relevant, 2 = slightly relevant, 1 = not relevant) was dichotomized (3 or 4 = relevant; 1 and 2 = not relevant). I-CVI was calculated by computing the number of expert-rated relevance divided by the total number of raters. Ideally I-CVI should not be less than 0.78 if there are 6 or more raters. Also, the range (maximum-minimum) for I-CVI would indicate more about the agreement between individual items. The S-CVI was calculated using the averaging method, where the average I-CVI was divided by the number of raters, as it reflects more on the averaging of the quality of item agreement rather than average rater’s performance. S-CVI should have a value of 0.8 at minimum [24].

*B. Structural validity:* judges the scoring coherence between scoring structure and the structure of the target domain. This is established by collecting evidence that reflects the correlation of the tool items and the tool scores [21]. In other words, collecting evidence that measures the ability of the (LMUP) items’ construct to measure planned pregnancy status as one item and to be correlated to the LMUP structure. Exploratory factor analysis (EFA) was used with the principal axis factoring (PAF) extraction method. In EFA, if items load on one factor (eigenvalue >1) this means that we are measuring one factor, which is the planning status of pregnancy (LMUP). The underlying latent structure would be explained by the extracted shared variance. Also, the strength of individual item relatedness to the scale in factor matrix was measured. Each item in the factor matrix ranges from 0 to 1. The closer the item to 1, the stronger the relatedness to the measured scale [25]. When the score nears 0, this can mean that 1) the item is too easy or too difficult to be answered; “item endorsement” should be > 10% and < 80%, or 2) it may not measure the target construct. Additionally, item communality in the EFA established the data’s strength in the factor analysis (i.e. showing unified high communalities without cross item loading among factor analysis); it also showed strong variables loading on each factor(s). Communalities are essentially item correlation, with >.8 meaning high and between .7 to > .4 reflecting moderate to low communality. If an item scored < .32, the item may not be related to the rest of items in the scale, or more factor exploration is recommended [26].

*C. Substantive validity:* examines the ability of the tool (LMUP) to reflect literature evidence or a theoretical framework coherent with attitudes of the target population [23]. This was established by hypothesis testing. Hypothesis testing is determining whether the respondent score is compatible with the literature evidence regarding the planning of a pregnancy. We tested two hypotheses: 1) that highly educated women tend to plan for pregnancy [27], i.e. they have high LMUP scores, and 2) that women with higher parity tend to report their pregnancy as unwanted [28], i.e. they have low LMUP score. Kruskal-Wallis test was used to evaluate differences in median LMUP scores by education and parity.

*D. Generalizability:* is the ability of the scale to be generalized among different populations with variable characteristics and in different settings. Establishing generalizability is accomplished by evidence of contextual stability and reliability. Contextual stability is the ability of an instrument to achieve consistent results in different time periods and/or in different settings, which are called situational and cross-sectional consistency, respectively [21]. Situational consistency tests whether answering the tool at different pregnancy time points changes the score results. We assessed situational consistency of the LMUP by calculating linear weighted Kappa (the non-parametric equivalent for intra-class correlation coefficient) for the test-retest reliability. In the test-retest reliability, we asked the respondents to retake the questionnaire on any day the following week after the first administration. The anonymous data linkage between the first and second administration was done via deterministic data linkage, using unique identifiers, including: age, parity, level of education, residency, and region. We could not assess long-term reliability (i.e. to test if women in postpartum may change her LMUP score from unplanned to planned or to wanted pregnancy) due to time constraints. Kappa score was interpreted as follows: moderate agreement (scores 0.4 - < 0.6), substantial agreement (scores 0.6- < 0.8), and almost perfect agreement (scores 0.8–1.00) [29].

Cross-sectional consistency tested whether women in different settings, i.e. residing in urban or rural areas, would have different score distribution. We assessed cross-sectional consistency by testing the difference of the LMUP score between urban and rural settings. Mann-Whitney U (the non-parametric equivalent of t-test) tests the median change of the LMUP score, between the two-residency locations.

Finally, reliability represents the tool’s ability to give a constant outcome every time. That was done by assessing Cronbach’s alpha statistics, which assumes that the average correlation of the set of items was a good estimate of the scale we were trying to measure. Cronbach’s alpha (> 0.7) is deemed acceptable.

Statistical analyses were carried out using IBM SPSS Statistics Grad Pack 24 for Mac (SPSS Inc.: *SPSS for Mac*, Version 24).

## Results

### Main sample and retest sample characteristics

The main sample had a total of 962 respondents, out of which 796 were included in the analysis. The majority of the respondents were from the eastern province of Saudi Arabia (87%). Their median age was 32 years (IQR 28,38); most of them had at least a Bachelor degree level of education (65%); around 60% were not pregnant nor nursing; and the median number of children the women had at the time of the study was 2 (IQR 1,4). The distribution of the sample characteristics is shown in [Table 1].

**Table 1:** Difference in the London Measure of Unplanned Pregnancy (LMUP) score by characteristics of the study sample of Saudi women.

	N (%)	Median LMUP (IQR)	P value
Residence			0.461*
Urban	606 (76)	8 (3,11)	
Rural	190 (24)	8 (4,11)	
Pregnancy/ nursing status			0.146**
Pregnant	107 (13)	9 (4,11)	
Nursing/ postpartum	214 (27)	8 (4,11)	
Not pregnant nor nursing	475 (60)	10 (6,12)	
Parity			<0.0001**
Currently pregnant with the first child	18 (2)	10 (6,12)	
1 child	186 (23)	10 (4,11)	
2 children	222 (28)	8 (4,11)	
3 children	159 (20)	6 (3,10)	
4 children	122 (15)	6 (2,10)	
5 children	47 (6)	5 (2,9)	
6 or more children	37 (5)	4 (2,7.5)	
Age (years)			0.352**
20–24	66 (8)	9 (5,11)	
25–29	179 (22)	8 (4,11)	
30–34	259 (33)	9 (4,11)	
35–39	145 (18)	8 (3,11)	
40 and more	147 (18)	8 (3,10)	
Educational attainment			0.011**
Primary school	6 (1)	9.5 (4.25,10.50)	
Intermediate school	10 (1)	8 (5.75,10.25)	
Secondary school	149 (19)	7 (3,10)	
Diploma	111 (14)	7 (4,10)	
Bachelor	422 (53)	8 (4,11)	
Post graduate	99 (12)	10 (5,11)	

* P-value obtained via Mann-Whitney test.

** P-value obtained via Kruskal-Wallis test.

For the retest sample, the majority of women resided in the eastern province (95%), two thirds had at least a bachelor degree level of education, 25% were pregnant and most of the pregnant women were in the last trimester, the median age of the last child was 3 years (IQR 1.8,4.9), and the median number of children was 2 years (IQR 1.5,3). On average, they sent the second response 5.4-days after the first response.

### LMUP score

**Fig 3:**
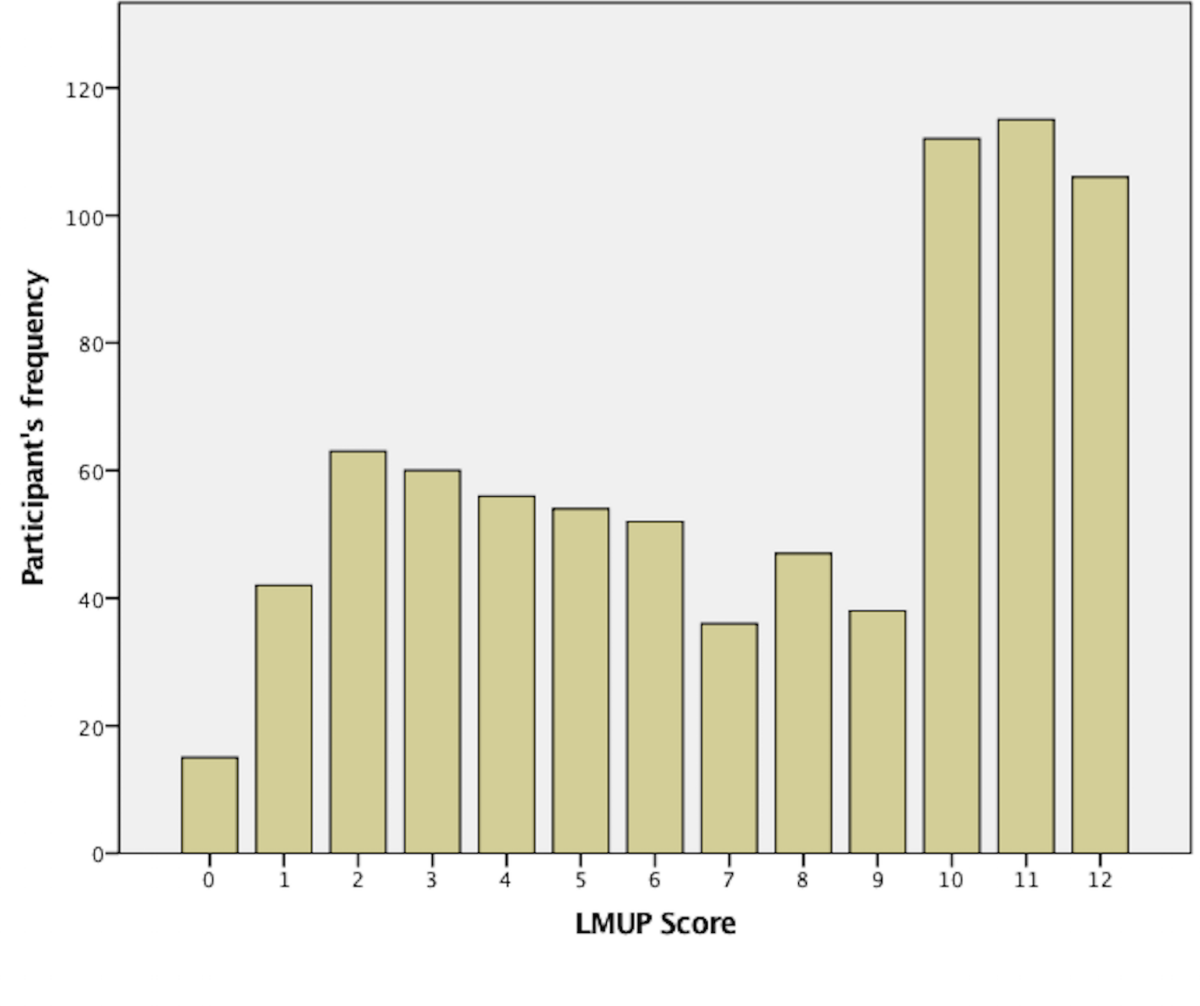
Distribution of the London Measure of Unplanned Pregnancy (LMUP) score for ever-married Saudi women. **LMUP score.** 0 unplanned pregnancy, 12 planned pregnancy.

The distribution of LMUP scores is shown in Fig 3. The median score was 8 (IQR 4,11). Twenty three percent (n = 180) of the participants had unplanned pregnancy in the last or the current pregnancy (LMUP score 0–3), 36% (n = 283) were in the ambivalent group (LMUP score 4–9), and 42% (n = 333) had their pregnancy planned (LMUP score 10–12). The distribution of the LMUP score according to the sample characteristics is shown in [Table 1].

### Psychometric properties

*A. Content validity:* The scale’s content appropriateness was confirmed by experts rating, 10 family planning practitioners with 3.5 median year of experience and (IQR 2,12). The content validity index was acceptable for both individual items (I-CVI > 0.78) and the overall scale (S-CVI > 0.8). The I-CVI range (maximum-minimum) was 0.4, reflecting the low index for the partner discussion item which was rated as not relevant by 4 out of the10 raters [Table 2].

**Table 2:** Expert rating of the six items in the Arabic version of London Measure of Unplanned Pregnancy (LMUP).

Experts Items/	1	2	3	4	5	6	7	8	9	10	Total agreement	Item CVI
Contraceptives	1	1	1	1	1	1	1	1	1	1	10	1.0
Timing	1	1	1	1	1	1	1	1	1	1	10	1.0
Intention	0	1	1	0	1	1	1	1	1	1	8	0.8
Desire	0	1	1	1	1	1	1	1	1	1	9	0.9
Partner	0	1	1	0	0	1	0	1	1	1	6	0.6
Preparation	1	1	1	1	1	1	0	1	1	1	9	0.9
Expert’s proportion	0.5	1.00	1.00	0.67	0.83	1.00	0.67	1.00	1.00	1.00	I-CVI = S-CV/Ave =	0.87 0.87

S-CVI/Ave, scale-level content validity index, averaging calculation method.

I-CVI, item-level content validity index

*B. Structural validity:* The data was suited for EFA based on tests of sampling adequacy; the Kaiser-Meyer-Olkin (KMO) test had a value of 0.885, and the Bartlett’s for Sphericity was statistically significant with a p-value <0.0001. Principal axis factoring for factors extraction confirmed that we extracted only one factor with all items in the scale loaded on 3.779 eigenvalue. The total shared variance was 63% for the extracted factor. Also, the factor matrix confirmed items correlation with the measured scale, with contraception and preparation having lower correlation [Table 3]. Furthermore, investigating item endorsement revealed that all items met the endorsement criteria, except for contraceptive which had less than 10% observation, representing women who were using contraceptive before the last pregnancy [Table 4].

**Table 3:** Reliability testing and exploratory factor analysis.

Factor Matrix* -(EFA)	Factor 1	Factor Communalities	Cronbach's alpha
			.878
Contraception	.544	.296	
Timing	.804	.712	
Intention	.930	.842	
Desire	.889	.803	
Partner	.778	.686	
Preparation	.482	.331	

*Extraction Method: Principal Axis Factoring. 1 factor extracted. 6 iterations required.

**Table 4:** Item endorsement for London Measure of Unplanned Pregnancy (LMUP) items.

Items	Categories	Frequency	Percent
Contraceptive use	0. Always using contraception	69	8.7
	1. Non-consistent use, or failed at least once	206	25.9
	2. Not using contraception	521	65.5
Timing	0. Wrong time	145	18.2
	1. Ok, but not quite the right time	240	30.2
	2. Right time	411	51.6
Intention	0. Did not intend pregnancy	262	32.9
	1. Intentions kept changing	154	19.3
	2. Intended pregnancy	380	47.7
Desire	0. Did not want baby	188	23.6
	1. Mixed feelings about having baby	204	25.6
	2. Wanted baby	404	50.8
Partner discussion	0. Never discussed having children together	194	24.4
	1. Discussed but I did not agree to get pregnant	188	23.6
	2. Discussed, and agreed to be pregnant	414	52.0
Preparation	0. Did no preparatory behaviors	426	53.5
	1. Did 1 preparatory behavior	211	26.5
	2. Did 2 or more preparatory behaviors	159	20.0
	Total	796	100.0

Factor communalities showed strong data, that the factor analysis demonstrated with unified loading on one factor only, and there was no cross loading among other factors. The strength of the data was shown as the following: intention and desire had a high correlation to other items in the scale; timing and partner had moderate communality, and contraceptive use and preparation had low communality [Table 3]. We conducted multiple factor extraction models for further factor exploration to investigate the relationship of the lower communality items. However, all types of extraction models (analysis not shown) resulted in one factor extraction, which confirms that the scale items are measuring one factor, “LMUP” i.e. unplanned pregnancy. The large sample size allowed for valid interpretation of the factors structure and inclusion of the items in the scale [26].

**Fig 4:**
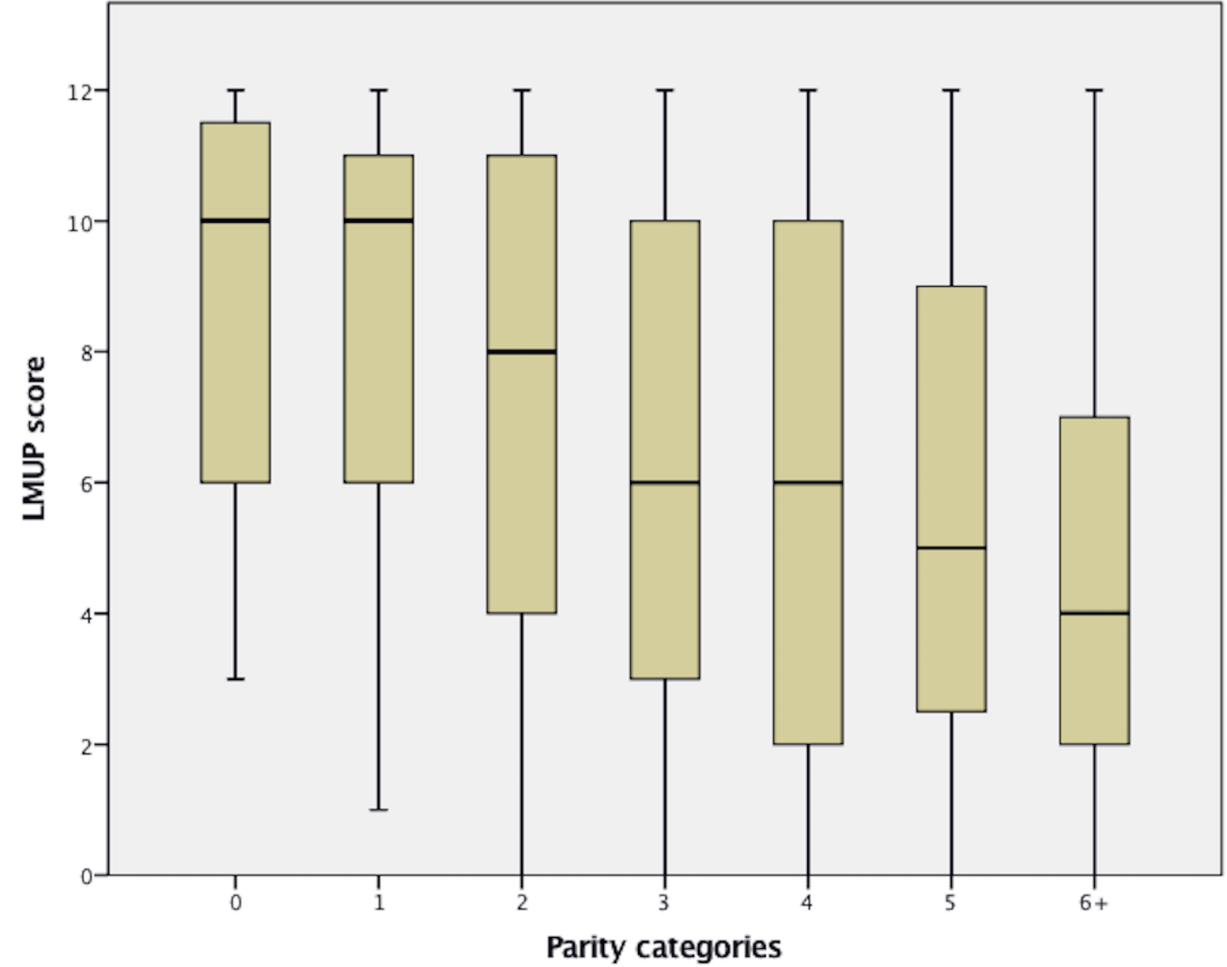
Distribution of London Measure of Unplanned Pregnancy (LMUP) score of Saudi women parity. 0 parity consisted of women who are had no prior born child, and/or currently pregnant with the first one.

*C. Substantive aspect of validity:* Hypothesis testing was met for both hypotheses. For the first hypothesis high education attainment was associated with a higher LMUP score (bimodal relationship). For the second hypothesis, a trend for lower LMUP score with higher parity was observed Fig 4.

*D. Generalizability aspect of validity:* Stability: We had substantial agreement as measured by linear weighted kappa (0.776) in the test-retest reliability. There was no significant difference in the median LMUP score between the first (median = 5.5; IQR 3.25–9.75) and the second administration (median = 5; IQR 4–10.75; p-value <0.0001). Cross-sectional stability for LMUP scores was established with both rural and urban areas having almost the same median score distribution and interquartile range of (Rural: median = 8, IQR 3,11; and Urban: median = 7, IQR 4,11; p-value = 0.461) [Table 1].

Reliability: Cronbach alpha test was above acceptable [Table 3].

## Discussion

This study translated the LMUP to Arabic, and validated it among Arabic-speaking Saudi women. Our study results provided evidence of validity and reliability of the Arabic LMUP.

The LMUP is a well-validated tool that measures intention/planning for pregnancy. It captures behaviors and attitudes that have been associated with pregnancy planning. Tools used to measure unintended pregnancy have been developed and applied mostly outside the Arabic world, where they address specific needs and desired outcomes (such as lowering the number of unintended pregnancies and unsafe abortions), aspects that may be different or inapplicable in an Arabic context. Furthermore, these tools are not sensitive to different contexts where, for example, contraception use is low and pregnancy planning is not as important in a family’s reproductive goals, apart from ensuring a reasonable spacing of around 24 months between pregnancies. In a previous evaluation of DHS data from 30 countries, a wide discrepancy in unwanted and mistimed pregnancy statistics was illuminated, and part of this discrepancy was attributed to statistics not being representative of actual values and circumstances due to the use of unstandardized translated tools, which might also be inappropriate for a population whose attitudes and beliefs on pregnancy have a complex, socially-embedded dimension [30]. Therefore, while proper tools that measure pregnancy planning behaviors by a multi-item questionnaire do exist, they have not been translated to Arabic and validated, leaving a gap in reproductive health data that is essential for policy decisions.

Arabic and Islamic cultures show gratitude and spiritual delight for a pregnancy. Birth control is considered acceptable primarily to space births to protect the health of the mother and alleviate the burden of frequent pregnancy on her and the rest of the family [31–33]. These feelings toward pregnancy shape women’s behavior regarding birth control use, which may explain the low contraceptive use and knowledge in countries such as Saudi Arabia [27, 34–36].

Our results provided evidence of technical validity and face validity of the Arabic version of LMUP, thereby confirming its content validity. However, there were some discrepancies between the specialists’ rating and the respondents’ LMUP score regarding the partner discussion item in the LMUP, which might be explained by two theories. The first is that the wording of the partner discussion item was confusing in the tool. For instance, the question asks the participant in the beginning about the circumstances around the current or most recent pregnancy, but then when the participant is asked whether or not they discussed having children together with their partner, the item choice listed “we *never* discussed having children together”. The second theory on this discrepancy is that couples might have discussed this and agreed on it a long time before pregnancy occurs, as observed in the UK [12]. Similarly, discussions of family size may occur at the beginning of a relationship, then subsequent discussion may follow according to the partner’s health and life constraints. We suggest future wording modification for the partner item for a consistent clear measurement of partner discussion.

Factor analysis established that all LMUP items were extracted as one factor. However, we had low communalities for preparation and contraceptive use. Therefore, we further evaluated item difficulty. All items in the scale achieved the desired criteria of item difficulty similar to what was found in the UK [28] and the US for both English and Spanish speakers [29]; all items had ≤80% observations. However, our study had contraceptive use <10% observation and had lower contraceptive item variability (Var. = 0.42) as 66% of the respondents were not using contraception before their latest pregnancy (contraception score 2) [Table 4.]. This might be explained by the fact that Saudi women had a low prevalence of contraceptive use of around 29% [16].

Hypothesis testing established substantive validity for the Arabic LMUP. Similar to the original LMUP in UK [28] and to the translated versions as in India [37], Malawi [38], and some other developing countries [10], our study found that women with higher parity are more likely than women with low parity to report their latest pregnancy as unplanned.

Though the sample had almost the same urban /rural distribution as the population level (76% urban and 24% rural), our study did not find a statistically significant difference in LMUP scores between women who live in rural and urban settings. This finding might be due to different factors, one being that contraceptive accessibility might be the same in both settings, as the Saudi Health care system services are free for citizens and most contraception is accessible over the counter [35]. In addition, the distribution of educational levels is nearly the same in both settings, which seem to influence pregnancy planning behavior [16, 35].

In this study’s validation process, it is important to highlight the consequential aspect of validity whereby the output score of the validated tool should be interpreted cautiously [21]. For example, when measuring pregnancy planning, women who have no concern about the “right” timing or the number of children they have, and who also accept their pregnancy as God’s will, should be considered fatalistic (believing in fate), rather than counting these pregnancies as unplanned or ambivalent [3]. For the case of fatalistic women, they believe there is no need for planning in general, and therefore, when they have unplanned pregnancies, they do not perceive the negative consequences related to their lives, pregnancy and birth. Our study may have included a few women who adopt this belief system, as revealed in replies such as: “I did not plan but I got pregnant and I’m happy “الحمدلله”/ “thank God!” It should be noted that this perception of pregnancy was also noticed in settings other than Saudi Arabia [35], such as Latina and non-Hispanic white women in Texas [27], and in white and African-American women in Pennsylvania [39]. Further studies should evaluate whether or not to categorize these attitudes as risky and whether they could result in negative maternal and fetal outcomes.

The most important strength of our study is that it is the first to validate and utilize a tool that measures pregnancy planning in Arabic. This tool will facilitate the investigation of circumstances around pregnancy planning in Arabic-speaking contexts and will enable the study of consequences of unplanned pregnancy. Furthermore, it will help identify women-at-risk and inform policy makers and family planning programs, thereby helping to improve maternal health in the Arab world. The pilot and the data collection of the LMUP in Arabic was determined acceptable for multiple reasons. First, the unexpectedly large sample size that received in a relatively short period of time allowed us to better examine stratified analysis by parity, education and urban-rural residence. Second, no critiques or unfavorable comments were received from study participants. Also, there were no missing data from LMUP items for individuals who could complete the online questionnaire.

Another strength of this study is that it was the first validation of LMUP tool to use self-administered electronic data collection using social media platforms. The use of electronic data collection eased the data collection burden and costs on researchers. Also, electronic collection allowed women to answer the survey in the setting of their choice, allowing them to feel more at ease while sharing their pregnancy planning behaviors. It also allowed them to have as much-needed time for introspection. Also, this study may have avoided social norm bias by introducing electronic data collection. Therefore, participants may have felt more comfortable sharing sensitive information.

Our study had some limitations. First, the number of test-retest participants (N = 24) was low, accounting for only 2.5% of the original sample (N = 796). With this small number, it is difficult to establish stability (i.e. weighted kappa) for all participants. Another limitation of the sampling methodology is the use of convenience non-probability sampling, which limits the generalizability of LMUP patterns to the overall population. However, compared to findings from other studies based on probability samples, our convenience sample was found to have similar validity and reliability. Also, our sample consisted of highly educated participants (65% had at least a bachelor degree), which is not representative of the education levels in the Eastern Province where the corresponding estimate is 16% [16]. While having a sample of highly educated women may have resulted in an underestimation of unplanned pregnancy prevalence [7], it is beyond this study’s objectives to measure the pregnancy planning rate. Moreover, we investigated whether the introduction of Arabic “blessing” term would change women’s responses to the questionnaire. And found that the word did not affect women responses; in fact, 0 women responded by “I want to have a baby” and had LMUP score 0–3. Also, 0 women responded by “I didn’t want to have a baby, had a total 7–12 LMUP score -analysis not shown-.

Furthermore, the electronic data collection method introduced some limitations. For example, the study could not calculate the participation rate and the sample is limited only to people who have internet access. Also, we did not ascertain the pregnancy outcomes of the latest pregnancy as the US LMUP study did [29], and participants who ended their pregnancy with abortion might be less likely to report or participate in the study than women who had a live birth outcome. This might underestimate the frequency of unplanned or unwanted pregnancy. However, we had one participant who reported her pregnancy outcome in the preparation item by “I lost latest pregnancy, I didn’t know I was pregnant” and other one said: “I had severe depression to the degree I wanted to have abortion”. This illuminates the tool’s ability to capture pregnancy outcomes in some instances.

In conclusion, this study provides evidence that the Arabic version of the LMUP is valid and reliable according to well-known psychometric criteria [21, 22, 24, 40]. This LMUP version can be used with confidence in research studies as a measure of unplanned pregnancy in Arabic settings. The translation was done based on formal Arabic and therefore can be easily understood in any Arabic setting. Future research is needed to evaluate the association of pregnancy planning and its health outcomes in the Arabic context.

## Supporting information

S1 Table Pregnancy intention measuring tools. *for clear up-to-date reference with specific modifications, see survey websites, as this information is beyond this study’s purpose.
(PDF)

S1 File Arabic version of LMUP. (PDF)

S2 File English back translation of LMUP. (PDF)

S3 File Piloting of the Arabic London Measure of Unplanned Pregnancy (LMUP) and words modification. (DOCX)

S4 File The validation dataset. (XLSX)
