# Supplementary material for: Validation of a pregnancy planning measure for Arabic-speaking women
Source: PLoS One. 2017 Oct 23;12(10):e0185433. doi: 10.1371/journal.pone.0185433 (PMC5653179; doi:10.1371/journal.pone.0185433)
Supplement: S1 Table — *for clear up-to-date reference with specific modifications, see survey websites, as this information is beyond this study’s purpose. (PDF) [file pone.0185433.s001.pdf]

Year	Question	Categories	Interpretation
**National survey of family growth (NSFG) [3]**			
1973-2016	1- Before you became pregnant . . . had you stopped using all methods of birth control?	1- Yes, No<br><br>(If Yes, go to 2; If No, if no go to 3)	
	2- Was the reason you had stopped using any methods because you yourself wanted to become pregnant?	2- Yes, No, I don't know (If yes, go to 5; If No, go to 6; If I don't know, go to 4)	
	3- At the time you became pregnant . . . did you yourself actually want to have a(nother) baby at some time?	3- Yes, No	3-Yes= Wanted<br>No= Unwanted
	4- It is sometimes difficult to recall these things, but just before that pregnancy began, would you say you probably wanted a(nother) baby at some time or probably not?	4- Probably yes, Probably no, Didn't care<br>(If probably yes, go to5; if probably no or didn't care, go to 6)	4- Probably no = Unwanted
	5- Did you become pregnant sooner than you wanted, later than you wanted, or at about the right time?	5- Sooner, Later, Right time, or Didn't care	5- Sooner= Mistimed

1995-2016	6- Happiness measurement: asked the respondent who happy show was when she know she was pregnant	6- A 10-point scale ranging from very happy to very unhappy	-Right time, later, didn't care= Wanted
	7- Ambivalent measurement: asked the respondent about her feelings around here pregnancy: e.g.	7- A 10-point scale ranging from strongly agree to strongly disagree e.g. Strongly agree	7- Maximum ambivalence
**Demographic and health survey (DHS) [10] in more than 90 countries**			
1948-2016	"At the time, you became pregnant with [name of last-born child], did you want to become pregnant then, did you want to wait until later, or did you want no more children at all?"	"I wanted to get pregnant then"	Intended
		"I wanted to get pregnant later"	Mistimed
		"I did not want any more children"	Unwanted
**Pregnancy Risk Monitoring Assessment System (PRAMS) by CDC [10]**			
1987-2016	"Thinking back to just before you got pregnant, how did you feel about getting pregnant?''	''I didn't want to be pregnant then or at any time in the future, or I wanted to be pregnant later.''	Unintended
		''I wanted to be pregnant, sooner, or I wanted to be pregnant then''	Intended
**Cross-sectional survey data from Indonesia [10]**			
1996	"Ever experience unintended pregnancy"	"Subjective (respondent report)"	Ever vs. never had unintended pregnancy
**Contraceptive Use, Pregnancy Intention and Decisions (CUPID) [17]**			
2011-2014	"Have you ever become pregnant by accident?"	Yes	Unintended
