# Supplementary material for: Validation of a pregnancy planning measure for Arabic-speaking women
Source: PLoS One. 2017 Oct 23;12(10):e0185433. doi: 10.1371/journal.pone.0185433 (PMC5653179; doi:10.1371/journal.pone.0185433)
Supplement: S1 File — (PDF) [file pone.0185433.s002.pdf]

الظروف المحيطة بالحمل

ادناه بعض الأسئلة حول الظروف المحيطة بالحمل وشعورك بتوقيت حدوث الحمل. **نرجو وضع الحمل الحالي (أو آخر حمل)** بالاعتبار عند الإجابة على الأسئلة.
الرجاء اختيار العبارة الأنسب في كل من الأسئلة التالية:

1) في الشهر الذي اصبحت به حاملا، انا وشريكي ....
- ☐ لم نستخدم مانع للحمل
- ☐ كنا نستخدم مانع للحمل، لكن ليس في كل الممارسات الحميمية.
- ☐ دائما نستخدم مانع للحمل، لكنني أعلم ان المانع فشل (تشقق، تحرك من مكانه، انخلع عن مكانه، لم يعمل, إلخ)
- ☐ دائما نستخدم مانع للحمل

2) أشعر أن حدوث حملي كان ......
- ☐ في الوقت المناسب
- ☐ جيد، لكنه ليس في الوقت المناسب تماما
- ☐ في وقت غير مناسب

3) تماما قبل حدوث الحمل
- ☐ كنت أنوي أن أحمل
- ☐ نيتي بالحمل كانت متذبذبة
- ☐ لم أكن أنوي أن أحمل

4) تماما قبل حدوث الحمل
- ☐ كنت أرغب أن أرزق بطفل
- ☐ كانت لدي مشاعر مختلطة بأن أرزق بطفل
- ☐ لم أكن أرغب أن أرزق بطفل

في السؤال التالي، المتعلق حول شريكك- ...

5) قبل حدوث الحمل
- ☐ أنا وشريكي كنا متفقين على أن أصبح حاملا
- ☐ أنا وشريكي تناقشنا على أن ننجب أطفالا ما بيننا، ولكن لم أوافق أن أصبح حاملا
- ☐ لم نناقش مطلقا أن ننجب أطفالا فيما بيننا

6) قبل حدوث الحمل، هل قمتي بأي شيء لتحسين صحتك لتهيئتك للحمل؟
(الرجاء اختيار كل ما هو مناسب من العبارات)
- ☐ أخذت حمض الفوليك
- ☐ توقفت أو قللت من التدخين، المعسل، او الشيشة
- ☐ توقف زوجي عن التدخين بوجودي او داخل المنزل
- ☐ أكلي أصبح صحيا أكثر.

- ☐ بحثت عن نصائح صحية/طبية
- ☐ اتخذت إجراء آخر، الرجاء الشرح________________

أو

- ☐ لم أفعل أي مما تم ذكره أعلاه قبل حملي.
