# Supplementary material for: Validation of a pregnancy planning measure for Arabic-speaking women
Source: PLoS One. 2017 Oct 23;12(10):e0185433. doi: 10.1371/journal.pone.0185433 (PMC5653179; doi:10.1371/journal.pone.0185433)
Supplement: S2 File — (PDF) [file pone.0185433.s003.pdf]

Eman Almaghaslah

Circumstances around pregnancy

Below some questions about the circumstances surrounding the pregnancy and the time you felt about it. Please put the current pregnant or the last one (Choose the most appropriate phrase)

11. In the month in which I became pregnant me and my partner...

- ○ we didn't use contraception
- ○ we have been using contraceptives but not at all intimate practices.
- ○ we always use contraceptives but I know it failed (crackling, moved from his place , dislocated from his place , it doesn't work , etc..)
- ○ we always use contraception

12. I feel that my pregnancy was happening...

- ○ In the suitable time
- ○ good , but not in suitable time
- ○ not suitable time

13. Completely before pregnancy

- ○ I intend to be pregnant
- ○ my intention was unsteady
- ○ I didn't intend to be pregnant

14. Completely before pregnancy

- ○ I wanted to have a baby
- ○ I had mixed feelings to have a baby
- ○ I didn’t want to have a baby

15. Before the pregnancy....

- ○ Me and my partner were agreed to become pregnant
- ○ we discussed to have children but , I didn't agree
- ○ we didn't discuss this issue

16. Before your pregnancy did you do anything to improve your health to prepare you ?

- ☐ I took Folic acid

- ☐ I stopped or reduced smoking
- ☐ My husband stopped smoking when I'm inside the house
- ☐ My Meals become healthier
- ☐ I searched for medical and health advices
- ☐ I didn't do anything from what is written above
- ☐ Other (please specify)

Thank you for filling the questionnaire, please refill this next week (after 7 days) if possible for once.
