# Supplementary material for: Validation of a pregnancy planning measure for Arabic-speaking women
Source: PLoS One. 2017 Oct 23;12(10):e0185433. doi: 10.1371/journal.pone.0185433 (PMC5653179; doi:10.1371/journal.pone.0185433)
Supplement: S3 File — (DOCX) [file pone.0185433.s004.docx]

**S3 File: Piloting of the Arabic London Measure of Unplanned Pregnancy (LMUP) and words modification:**

The pilot sample included 5 women. Their mean age was 32.4 + 2.2 years and all were from eastern province of Saudi Arabia. A few changes were made according to the results of the pretest. First, we altered the choice categories on pregnancy preparation item of the Arabic LMUP by removing “alcohol” and adding “partner smoking outside of the house” as a culturally acceptable measure for pregnancy preparation. Second, instructions for each question were summed up in the beginning of the tool as the first five question are multiple choice, then the instruction were retained for the last question to choose all that apply. Furthermore, we evaluated whether the use of “partner” as it appeared in the original LMUP was understandable as “husband”. This was confirmed, and accordingly no changes were made to the term. The last change was the introduction of the Arabic word “أُرْزَقْ” / ‘blessed’ in the desire item of LMUP that asks about desire to have a baby, as this is the term that is used in Arabic for the term “having a baby”. The LMUP back translation was consistent with the original English version.
